# The complete mitogenome, phylogenetic placement and *cox1* variation of Cape sea urchins (*Parechinus angulosus*) in southern Africa

**DOI:** 10.1007/s11033-026-12198-8

**Published:** 2026-07-01

**Authors:** Suzanne Redelinghuys, Arsalan Emami-Khoyi, Gwynneth Matcher, Peter R. Teske, Sándor Csányi, Miklós Heltai, Robert J. Toonen, Francesca Porri

**Affiliations:** 1 South African Institute for Aquatic Biodiversity, Makhanda, 6139 South Africa; 2 Department Zoology & Entomology, Rhodes University, Makhanda, 6140 South Africa; 3 Institute for Wildlife Management and Nature Conservation, Department of Wildlife Biology and Management, Hungarian University of Agriculture and Life Sciences, Gödöllő, 2100 Hungary; 4 Centre for Ecological Genomic and Wildlife Conservation, University of Johannesburg, Auckland Park 2006, Johannesburg, South Africa; 5 Department Biochemistry & Microbiology, Rhodes University, Makhanda, 6140 South Africa; 6 Hawai‘i Institute of Marine Biology, University of Hawai‘i at Mānoa, 46- 007 Lilipuna Road, Kāne‘ohe Hawai‘i, 96744 Kāne‘ohe, USA

**Keywords:** Mitogenome assembly, phylogenetics, ezRAD, Oxford Nanopore Technology, phylogeography, Cape sea urchin, *cox1* barcodes

## Abstract

**Background:**

The Cape sea urchin, *Parechinus angulosus*, is a widely distributed keystone species that inhabits intertidal and subtidal ecosystems along the South African coastline. Despite its importance as an ecosystem engineer, its phylogenetic placement and mitochondrial genomic (hereafter mitogenome) variation remain poorly understood. In the current study, we present the first complete mitogenome for this species, assembled from long-read sequences generated on the Oxford Nanopore sequencing platform, and investigate its phylogenetic placement among other sea urchin species using a combination of Bayesian Inference and Maximum-Likelihood methods.

**Methods and results:**

A circular genome of 15 722 bp, with an average coverage of 159, comprising 13 protein-coding genes, two rRNAs and 22 tRNAs, was assembled *de novo*. Phylogenetic reconstructions based on 13 protein-coding genes recovered *Paracentrotus lividus* as the sister taxon of *P. angulosus*, and these two species formed a monophyletic clade with *Loxechinus albus* and *Sterechinus neumayeri* within Camaradont sea urchins. Using the mitogenome assembly as a template, an additional set of 29 *cox1* sequences was mined from publicly available genomic sequences. These revealed that Cape sea urchins maintain substantial mitogenomic variation across their distribution range, expressed predominantly as low-frequency haplotypes.

**Conclusion:**

This study demonstrates that the Cape sea urchin is genetically distinct within the order Camarodonta and exhibits considerable variation in the *cox1* gene across coastal habitats of southern Africa. Furthermore, the identification of a large number of low-frequency haplotypes may indicate population expansion or ongoing purifying selection.

**Supplementary Information:**

The online version contains supplementary material available at 10.1007/s11033-026-12198-8.

## Introduction

**Fig. 1:**
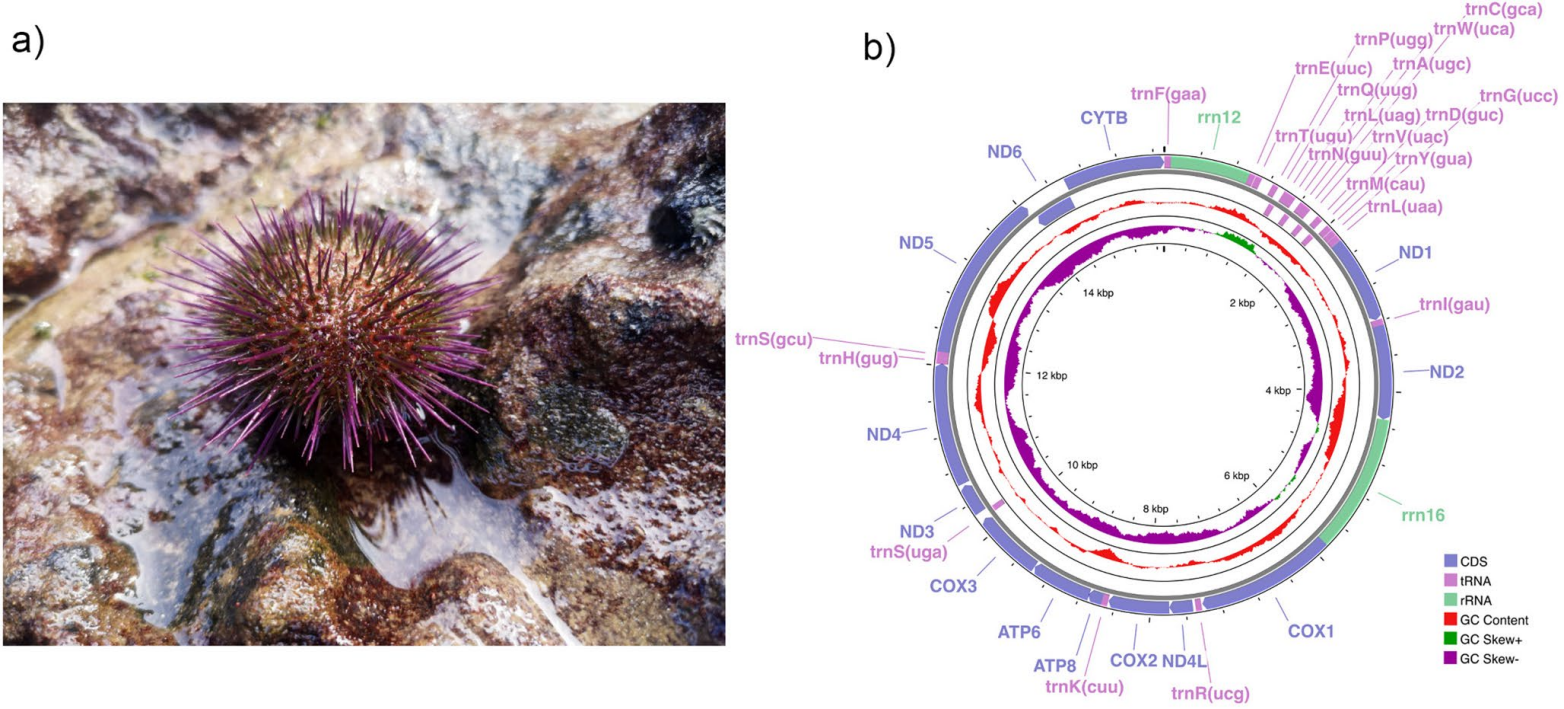
(A) The Cape sea urchin, *Parechinus angulosus* (Photo: C.I. Green 2024). (B) A graphical representation of the *Parechinus angulosus* mitogenome showing the location of 13 protein-coding genes, 22 tRNAs, and two rRNAs. The inner bars represent the GC content and GC skew (b). This figure was generated by the Proksee web server [13]

The South African coastline, spanning approximately 2 800 km, is home to 74 echinoid species. Among these, the Cape sea urchin, *Parechinus angulosus* (Leske, 1778) (Fig. 1) A, has one of the largest gseographical distributions among the region’s echinoid species, extending from Lüderitz Bay in Namibia to Umhlali Beach in northern KwaZulu-Natal, South Africa [1] across a diverse range of marine habitats. Earlier studies established a direct link between coastal water turbulence, substrate type, temperature, food availability, and predation on the species’ potential to establish itself in coastal habitats with contrasting marine features [2].

*Parechinus angulosus* is a medium-sized sea urchin that rarely reaches a maximum test diameter (the hard outer shell of the urchin) of 60 mm. It feeds on drift algae and on the early sporophyte stages of the kelp *Ecklonia maxima* [1]. The Cape sea urchin is known for modifying marine habitats and providing shelter and edible resources for other marine organisms, such as the juveniles of the commercially important abalone, *Haliotis midae* [3]. Despite the ecological importance of *P. angulosus* as a keystone ecosystem engineer, its life history remains poorly understood, and its phylogenetic relationships compared to other sea urchins require further investigation.

Mitogenomic markers remain the preferred choice for phylogenetic research and species identification, despite certain drawbacks [4–6]. In molecular methods designed to survey biodiversity across entire ecological communities, known as eDNA metabarcoding, reference databases of mitogenomic markers, such as *cox1*, are frequently used to assign taxonomic rank to sequences generated from these communities. In southern Africa, where particularly marine taxa lack reference sequences, the resulting difficulties in assigning taxonomic rank hinder the integration of molecular data into broader ecosystem management plans.

The present study makes two significant contributions towards molecular research on the Cape sea urchin. First, a complete mitogenome for this species was assembled *de novo*, annotated and described, using sequences generated with the Oxford Nanopore sequencing platform, followed by phylogenetic reconstruction to assess its placement among other sea urchins for which mitogenomes are available to date. Second, this template mitogenome was used as a reference to mine a reduced-representation genomic dataset from an additional 29 individuals, to report the first publically avialable *cox1* reference database for this species, and to assess the diversity of this gene across its distribution range in South Africa.

## Materials and methods

### Sample collection and DNA extraction

A specimen of *Parechinus angulosus* was collected by hand from an intertidal rock pool at Cannon Rocks (-33.75142, 26.54546) on the eastern south coast of South Africa. The collected specimen was preserved in 99% ethanol and stored at -27 °C at the South African Institute for Aquatic Biodiversity until processed. Genomic DNA was extracted from intestinal tissue using a modified salting out protocol [7], which included the addition of 10 µL of 20 ng/µL ribonuclease A (Glentham Life Sciences, UK) after the protein pelleting step. The extract was purified using Mag-Bind^®^ Total Pure NGS SPRI beads (Omega Bio-Tek Inc., USA) according to the manufacturer’s instructions.

### Oxford Nanopore library preparation and sequencing

Extracted genomic DNA was quantified using a Qubit^®^ 3.0 fluorometer with the Qubit™ dsDNA Quantification Assay Kit (Thermo Fisher Scientific, USA), and DNA purity was further assessed by measuring the A260/280 and A260/230 ratios using a NanoDrop 2000 spectrophotometer (Thermo Fisher Scientific). Prior to sequencing, the integrity of DNA fragments was visually evaluated by agarose gel electrophoresis (0.7% agarose) using λ HindIII DNA as a high-molecular-weight size marker. Following these steps, a genomic library was prepared from 1 µg of input gDNA using the Ligation Sequencing DNA Kit V14 (SQK-LSK114; Oxford Nanopore Technologies) according to the manufacturer’s protocol. Sequencing was performed on a PromethION 2 Solo (P2 Solo) platform using an R10.4.1 PromethION flow cell (FLO-PRO114M; Oxford Nanopore Technologies). Raw signal data were base-called using MinKNOW software v.24.11.10 (Oxford Nanopore Technologies) with the high-accuracy (HAC) base-calling model (v4.3.0, 400 bps). Library preparation and sequencing were conducted by the Aquatic Genomics Research Platform at the South African Institute for Aquatic Biodiversity (AGRP, NRF-SAIAB).

### Mitogenome assembly and annotation

The quality of Nanopore raw reads was visually assessed using FastQC (https://www.bioinformatics.babraham.ac.uk/projects/fastqc/). These reads were then assembled *de novo* using Flye v.2.9 assembler [8]. All parameters of the assembler were set to their default values, except the maximum contig size, which was set to 18 000 bp, consistent with the maximum expected mitogenome size in sea urchins. The circular contig with the highest coverage was then extracted and BLAST-searched against the NCBI nucleotide database to confirm its taxonomic origin. Following this step, this contig was annotated using a combination of MitoZ v.3.6 [9] and MITOS1 [10], and the boundaries of the mitogenomic features were manually adjusted by visual inspection of the start and stop codons reported for this group (NCBI codon Table 9) in Geneious Prime v.2024.0 [11]. Synonymous codon usage, GC and AT skews were calculated using the EZmito pipeline [12]. A graphical representation of the mitogenome was created using the Proksee web server (Fig. 1B) [13].

### Phylogenetic reconstruction

To reconstruct the phylogenetic placement of *Parechinus angulosus* among other sea urchins for which complete mitogenomes are available, protein-coding sequences of 45 closely related species (for full accession numbers, please see Supplementary Information, Table 1) were downloaded from the NCBI database. This subset was selected with particular attention to selecting at least one representative of each major sea urchin genus for which a complete mitogenome sequence is currently available. The sequences for each protein-coding gene were then separately aligned using MAFFT v.7 [14]. Prior to phylogenetic analysis, the highly variable third codon positions of each gene were removed.

A phylogenetic tree based on the available mitogenomes was reconstructed using Bayesian Inference (BI) and Maximum Likelihood (ML). Bayesian inference was conducted using BEAST2 v.2.7 [15]. The input file for the BEAST2 analysis was prepared in the accompanying package BEAUti v.2.7. To predict the best substitution model for each protein-coding gene, bModelTest v.1.3.3 [16] was used. The bModelTest averages across different substitution models; as such, the uncertainty in nucleotide substitution for each protein-coding gene can be accounted for during phylogenetic tree search. For the phylogenetic tree priors, a Yule speciation model was selected. The remaining parameters were set to the recommended default values. BEAST2 was run using 10 independent chains, each with 500 000 000 iterations and an initial burn-in of 99 000 000 steps. The resulting log files were visualised in Tracer v.1.7.2 [17], and convergence of the different chains was visually assessed. Following this step, the generated tree files were combined using LogCombiner v.1.10 [18], and a maximum clade consensus tree was generated in TreeAnnotator v.1.4 [19] and visualised in FigTree v.1.4.4 [20]. A Maximum Likelihood phylogenetic tree with 9999 fast bootstrap replications was reconstructed in RAxML-ng v.1.2 [21] by optimising parameters in a GTR [22] nucleotide substitution model for each partition using RaxML’s built-in approach. The consensus tree was similarly visualised in FigTree.

### Capturing mitochondrial *cox1* barcoding markers

Twenty-nine genomic DNA libraries generated using the ezRAD protocol [23], a simplified restriction site-associated DNA marker, were downloaded from the NCBI database. This genomic dataset was reported by Redelinghuys et al. [24] (Supplementary Information, Table 2). Sequences were assembled *de novo* using MEGAHIT v.1.2 [25], and all scaffolds of mitochondrial origin were identified by conducting BLAST searches against the NCBI database. These sequences were subsequently aligned against the reference *cox1* sequence, assembled and annotated from Oxford Nanopore sequences using the command-line version of MAFFT. All reliably aligned sequences were extracted and translated into their corresponding proteins to inspect for misplaced stop codons, which could indicate sequence artefacts or erroneous assembly. The *cox1* sequences were aligned using MAFFT v.7, and mitogenomic diversity in terms of θ_S_, θ_K_, and Tajima’s *D* was investigated using Arlequin v. 3.5 [26]. Furthermore, the ratio of non-synonymous to synonymous nucleotide substitutions (dN/dS) was calculated using the SALC method on the Datamonkey 2 server [27].

The correlation between geographical distance and Kimura’s two-parameter genetic distance(K2P) [28] was investigated using the isolation-by-distance (IBD) function in adegenet v.2.1 R package [29]. A Bayesian phylogenetic tree was then reconstructed from c*ox1* sequences using BEAST2, following the same steps as those described for the complete mitogenome, and superimposed across the landscape using the phytools v.2 R package [30].

## Results and Discussion

The Oxford Nanopore sequencing run produced 25 494 917 raw reads. The Flye assembler reconstructed a single 15 722 bp circular contig with an average coverage of 159 (NCBI accession number PX523763) and a GC content of 39.8%. The annotation pipelines identified a total of 37 mitogenomic features, including 13 protein-coding genes, two ribosomal RNAs (rRNAs), and 22 transfer RNAs (tRNAs) on the assembled contig (Fig. 1B). A blast search of NCBI nucleotide sequences recovered *Paracentrotus lividus* (NCBI accession number PP438737.1) as the most closely related species for which a complete mitogenome is currently available.

The most common start codon was ATG, and only *atp8* and *nad3* protein-coding genes started with GTG. Similarly, TAA and TAG were predicted to be the most common stop codons, with 9 and 4 occurrences, respectively. Several non-coding regions, with an average length of 11 bp, including a long fragment of approximately 134 bp, were also identified. Relative Synonymous Codon Usage (RSCU), GC content, and AT skew were similar to those reported in closely related species (Supplementary Information, Fig. 1).

**Fig. 2:**
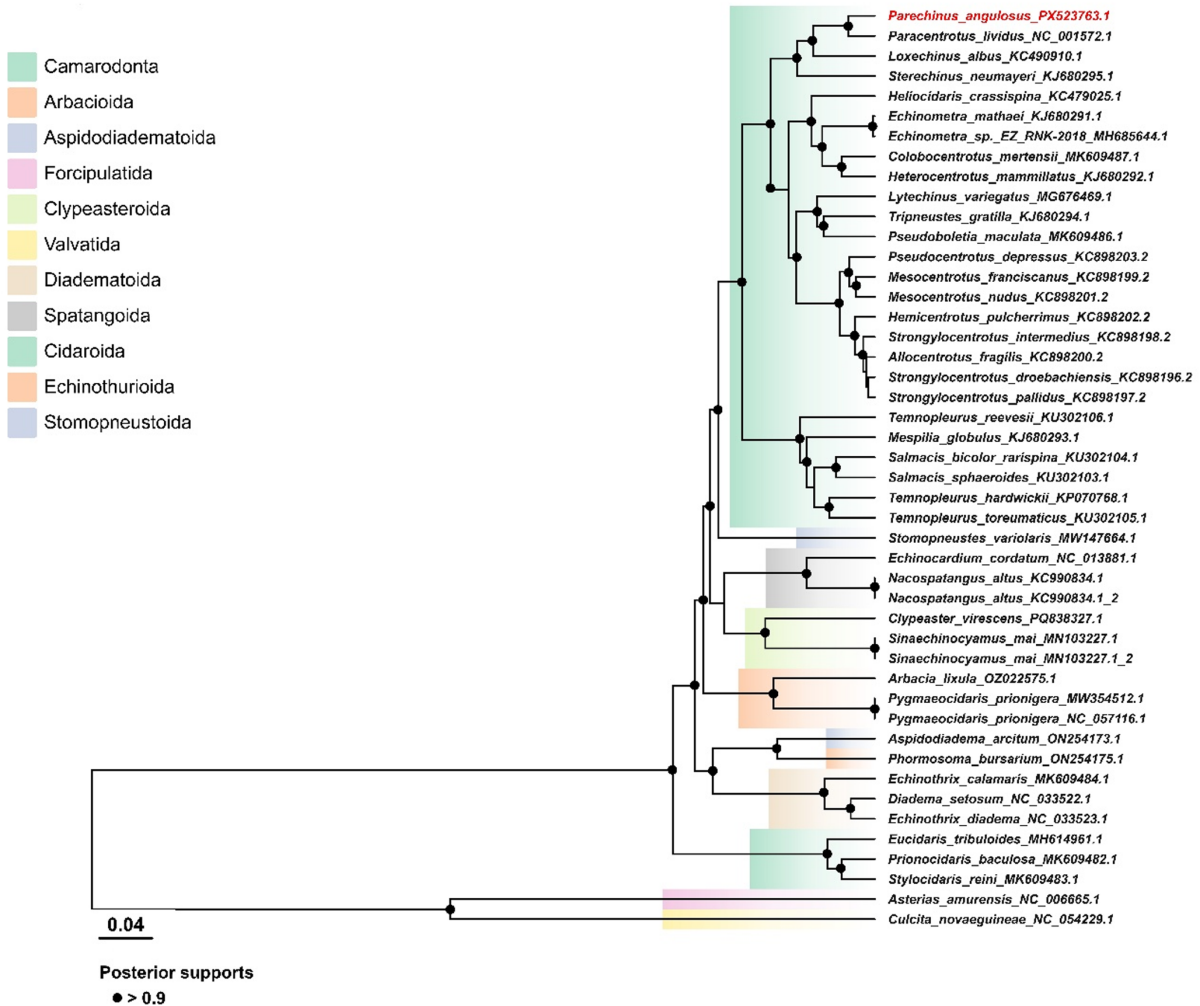
A Bayesian ultrametric phylogenetic tree reconstructed based on 13 protein-coding genes using BEAST2 showing the phylogenetic placement of *Parechinus angulosus* (highlighted in red) relative to other sea urchin species

**Fig. 3:**
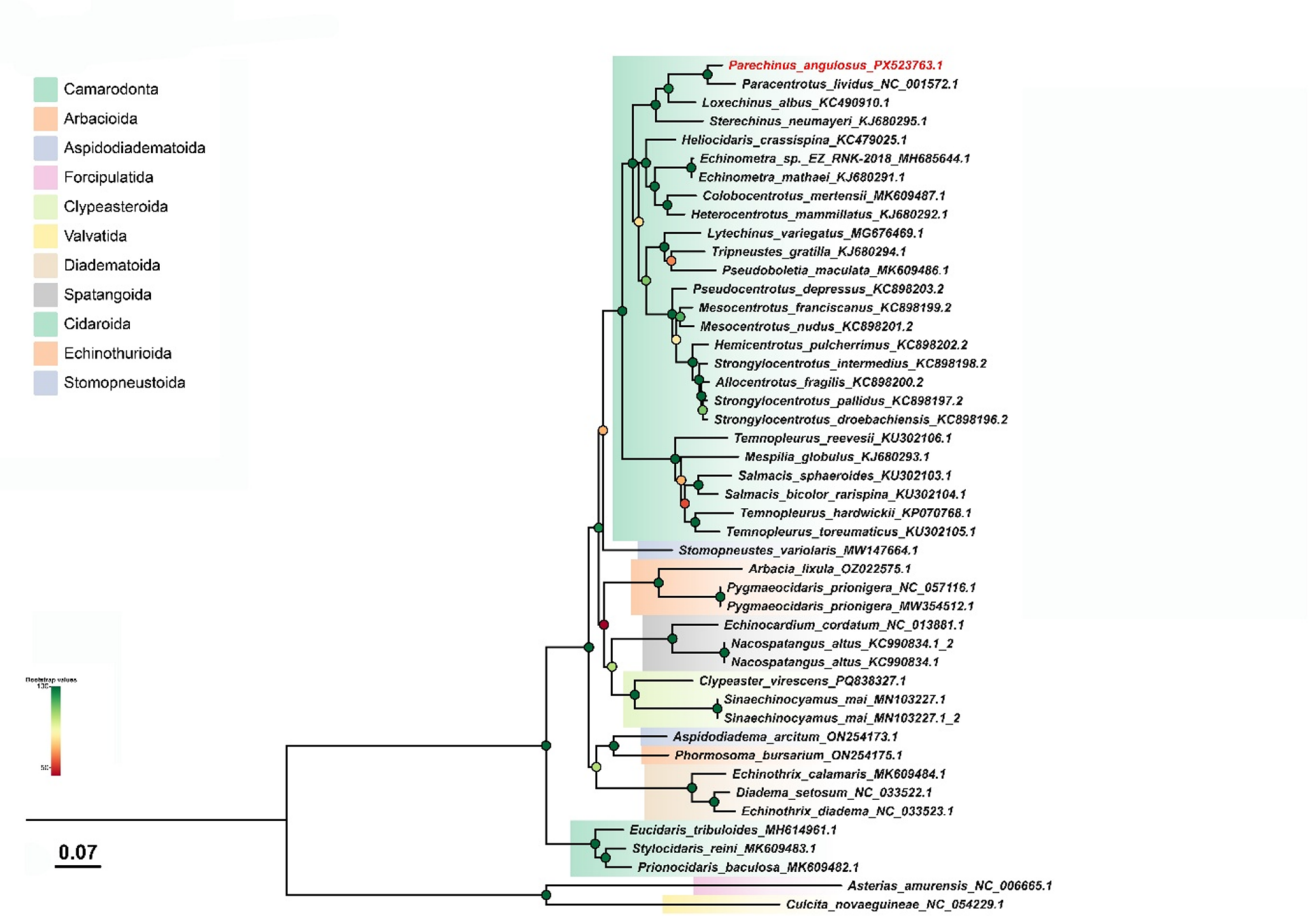
Maximum-likelihood phylogenetic tree reconstructed using RAxML-ng based on 13 protein-coding sequences showing the phylogenetic placement of *Parechinus angulosus* (highlighted in red) among other related sea urchin species. The colour bar on the left shows bootstrap supports

After removing the highly variable third codon position from the alignment of 13 protein-coding genes, 46 sequences each 7 665 bp long, including 3 312 variable sites and 2 792 parsimony-informative loci, were retained. In the Bayesian phylogenetic analysis, visual inspection of the trace files showed that all independent chains converged on a single consensus topology. The Effective Sample Size (ESS) for the estimated parameters was > 1 000, confirming that the number of iterations was sufficient to achieve convergence. The Bayesian phylogenetic analysis placed *P. angulosus* as a sister taxon of *Paracentrotus lividus* (Fig. 2). These two species formed a monophyletic group with *Loxechinus albus* and *Sterechinus neumayeri*. The topology of the Maximum Likelihood (ML) phylogenetic tree (Fig. 3) was identical to that of the Bayesian tree. The topology of both trees was consistent with earlier phylogenetic studies on echinoid mitogenomes across different orders [31].

The mining of publicly available c*ox1* sequences retrieved 29 sequences, with a mean length of 1 443 bp, a value close to the maximum length of 1 550 bp reported for sea urchins. In total, 21 unique haplotypes and 57 polymorphic sites were identified. Among these, 49 transitions and 8 transversions were observed. Pairwise nucleotide diversity, expressed as *θ*_*S*_ and *θ*_*K*_, was estimated at 14.51 and 6.29, respectively. Tajima’s *D* was − 2.01, consistent with population expansion or purifying or positive selection [32]. The estimated dN/dS ratio across the alignment was less than one, indicating that the *cox1* gene could be predominantly under purifying selection. A larger sample size than that used in this study, including nuclear markers, is required to reach a definitive conclusion, making this an interesting topic for future research.

**Fig. 4:**
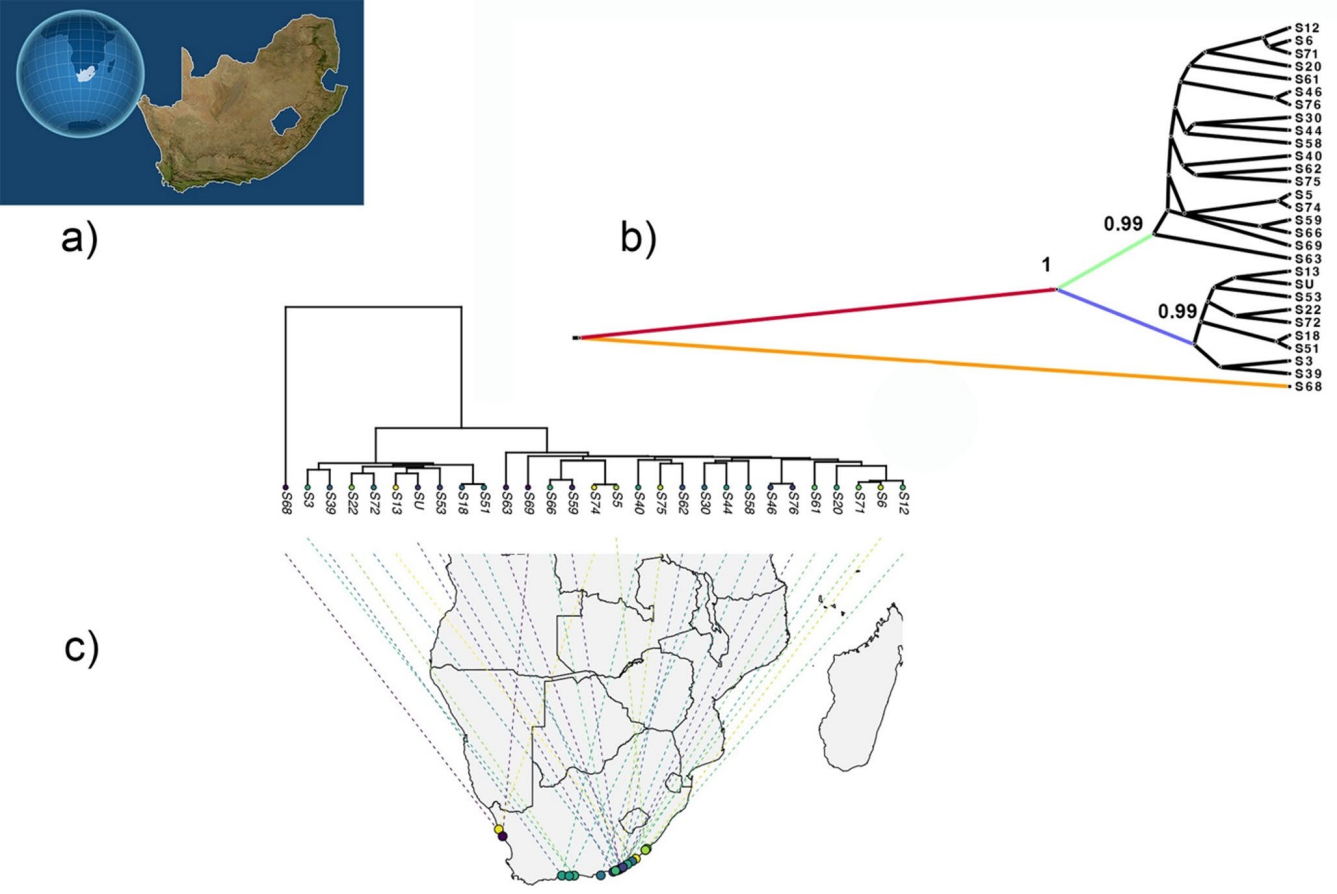
A map illustrating the geographical location of the current study (a). An ultrametric Bayesian phylogenetic tree reconstructed from 29 *cox1* sequences (b), and spatial distribution of haplotypes along the rocky shores of South Africa (c). The values on the phylogenetic tree show posterior probabilities for each split

The topology of the Bayesian *cox1* tree across the distribution range of this species reveals two major clades and a basal lineage represented by a single individual that split early from the most recent common ancestor of the above-mentioned clades (Fig. 4). Isolation-by-distance analysis revealed a significant positive correlation between K2P genetic and geographic distances (*r* = 0.42, p*-value =* 0.03), indicating that the distribution of *cox1* diversity along the coastline follows a non-random, distance-dependent pattern.

Mitogenomic markers have long been the first choice in phylogenetic and population genetic studies [33, 34]; however, inferences based on the mitogenomes have several important limitations that must be considered when interpreting results [35]. Maternally inherited mitogenomes represent a single realisation of the coalescent processes that occur throughout a species’ evolutionary history. The non-recombining nature of this locus means it may not completely reflect the evolutionary history of the study species or population, and its comparatively smaller effective population size further increases the risk of misleading phylogenetic inferences. Furthermore, hybridisation and mitogenomic introgression can potentially cause mitochondrial genealogy to diverge from the true species and population history [36–38]. Beyond these constraints, recent studies have shown that purifying and positive selection can act on mitochondrial protein-coding regions, violating the assumption of selective neutrality, which is fundamental to molecular clock analyses and dating divergence between species [39]. Despite these limitations, mitogenomic markers can serve as valuable stepping stones, providing preliminary insights into population structure and evolutionary history that can guide and inform future genome-wide investigations.

### Challenges and Technical limitations

Metagenomic assemblers such as MEGAHIT, which was used in this study to capture *cox1*, occasionally incorporate nuclear mitochondrial DNA segments (NUMT) or chimeric sequences into assemblies. Using a reference guide derived from long-read sequencing technologies, which are less prone to including such fragments, can help mitigate this issue. Additionally, capturing mitochondrial sequences from genomic data generated by restriction-site-associated DNA sequencing depends on the presence of sufficient cleavage sites throughout the mitogenome. Performing in *silico* digestion on the same or a closely related species can therefore help identify subsets of publicly available datasets likely to contain sufficient organelle-derived sequences.

## Conclusion

This study described the first complete mitogenome of the Cape sea urchin, an emerging model marine organism native to southern Africa, and contributed to mitogenomic resources for further study of African echinoderm evolution. The Cape sea urchin was recovered as a sister taxon to the northern hemisphere *Paracentrotus lividus*, and the two species formed a monophyletic group with the southern hemisphere species of *Loxechinus albus* and *Sterechinus neumayeri*. This also emphasises the lack of complete mitogenomes for southern-hemisphere echinoid species and offers a fruitful area for future mitogenomic research. The mined *cox1* sequences confirm that the population of Cape sea urchins harbours considerable mitogenomic diversity across its distribution area, mainly represented as low-frequency *cox1* haplotypes.

## Supplementary Information

Below is the link to the electronic supplementary material.

Supplementary Material 1

## Data Availability

The raw sequences generated for this project are available at the National Centre for Biotechnology Information under Bio Project PRJNA1098704. The assembled Mitogenome has been submitted to the same repository under the accession number PX523763.
