# Supplementary material for: The complete mitogenome, phylogenetic placement and cox1 variation of Cape sea urchins (Parechinus angulosus) in southern Africa
Source: Mol Biol Rep. 2026 Jul 1;53(1):1071. doi: 10.1007/s11033-026-12198-8 (PMC13323609; doi:10.1007/s11033-026-12198-8)
Supplement: Supplementary file 1 — Supplementary Material 1 [file 11033_2026_12198_MOESM1_ESM.docx]

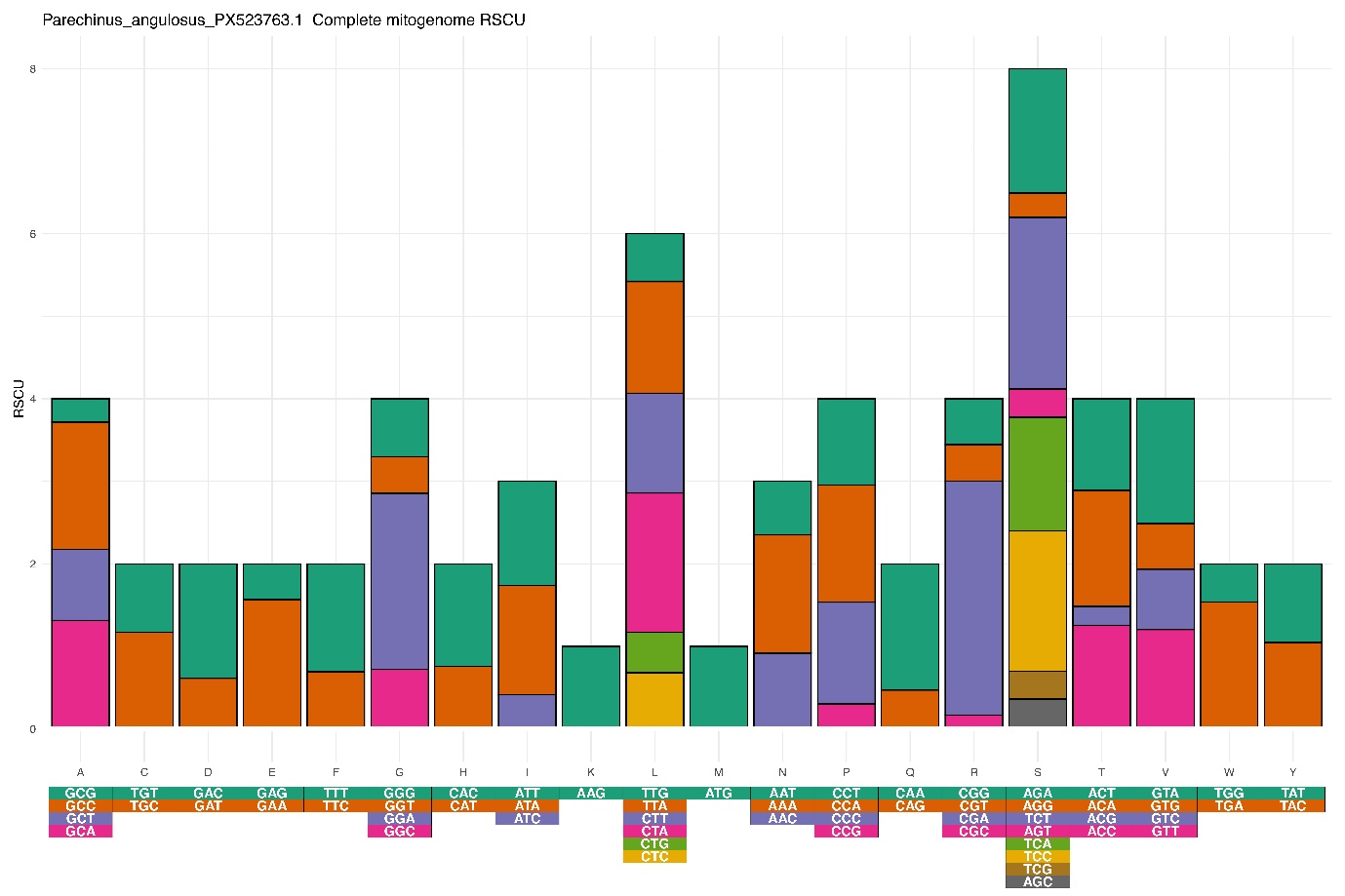


S.I. Figure 1: Relative Synonymous Codon Usage (RSCU) estimated from the 13 protein-coding genes in the *Parechinus angulosus* mitogenome. Different amino acids are represented by letters on the x-axis.


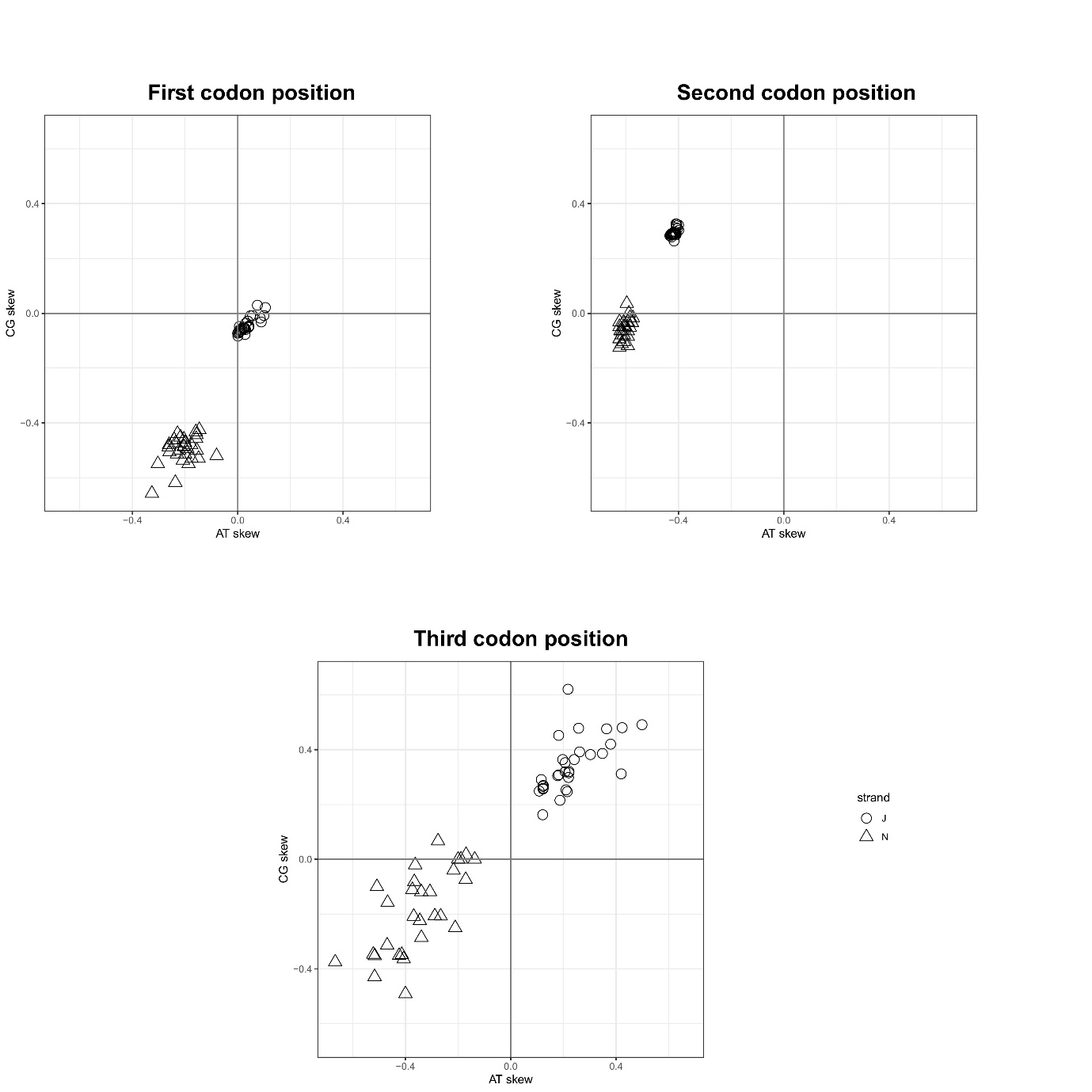


S.I. Figure 2: Relationship between AT skew and GC skew at first, second, and third codon positions for the genome of the Cape sea urchin, *Parechinus angulosus*.

S.I. Table 1: Accession numbers for sea urchin species included in the phylogenetic tree (Figures 4 and 5).

| Species | Accession number |
| --- | --- |
| *Allocentrotus fragilis* | KC898200.2 |
| *Arbacia lixula* | OZ022575.1 |
| *Aspidodiadema arcitum* | ON254173.1 |
| *Asterias amurensis* | NC_006665.1 |
| *Clypeaster virescens* | PQ838327.1 |
| *Colobocentrotus mertensii* | MK609487.1 |
| *Culcita novaeguineae* | NC_054229.1 |
| *Diadema setosum* | NC_033522.1 |
| *Echinocardium cordatum* | NC_013881.1 |
| *Echinometra mathaei* | KJ680291.1 |
| *Echinometra sp. EZ RNK-2018* | MH685644.1 |
| *Echinothrix calamaris* | MK609484.1 |
| *Echinothrix diadema* | NC_033523.1 |
| *Eucidaris tribuloides* | MH614961.1 |
| *Heliocidaris crassispina* | KC479025.1 |
| *Hemicentrotus pulcherrimus* | KC898202.2 |
| *Heterocentrotus mammillatus* | KJ680292.1 |
| *Loxechinus albus* | KC490910.1 |
| *Lytechinus variegatus* | MG676469.1 |
| *Mesocentrotus franciscanus* | KC898199.2 |
| *Mesocentrotus nudus* | KC898201.2 |
| *Mespilia globulus* | KJ680293.1 |
| *Nacospatangus altus* | KC990834.1 |
| *Nacospatangus altus* | KC990834.12 |
| *Paracentrotus lividus* | NC_001572.1 |
| *Parechinus angulosus* | PX523763.1 |
| *Phormosoma bursarium* | ON254175.1 |
| *Prionocidaris baculosa* | MK609482.1 |
| *Pseudoboletia maculata* | MK609486.1 |
| *Pseudocentrotus depressus* | KC898203.2 |
| *Pygmaeocidaris prionigera* | MW354512.1 |
| *Pygmaeocidaris prionigera* | NC_057116.1 |
| *Salmacis bicolor rarispina* | KU302104.1 |
| *Salmacis sphaeroides* | KU302103.1 |
| *Sinaechinocyamus mai* | MN103227.1 |
| *Sinaechinocyamus mai* | MN103227.1_2 |
| *Sterechinus neumayeri* | KJ680295.1 |
| *Stomopneustes variolaris* | MW147664.1 |
| *Strongylocentrotus droebachiensis* | KC898196.2 |
| *Strongylocentrotus intermedius* | KC898198.2 |
| *Strongylocentrotus pallidus* | KC898197.2 |
| *Stylocidaris reini* | MK609483.1 |
| *Temnopleurus hardwickii* | KP070768.1 |
| *Temnopleurus reevesii* | KU302106.1 |
| *Temnopleurus toreumaticus* | KU302105.1 |
| *Tripneustes gratilla* | KJ680294.1 |

S.I. Table 2: SRA accession numbers for NCBI Bioproject PRJNA1156167.

| Experiment accession number | Sample accession number | Sample ID |
| --- | --- | --- |
| SRX24215420 | SRS20985619 | S92 |
| SRX24215419 | SRS20985618 | S88 |
| SRX24215418 | SRS20985616 | S87 |
| SRX24215417 | SRS20985617 | S72 |
| SRX24215416 | SRS20985614 | S30 |
| SRX24215415 | SRS20985615 | S59 |
| SRX24215414 | SRS20985613 | S51 |
| SRX24215413 | SRS20985612 | S9 |
| SRX24215412 | SRS20985611 | S13 |
| SRX24215411 | SRS20985610 | S81 |
| SRX24215410 | SRS20985609 | S86 |
| SRX24215409 | SRS20985607 | S62 |
| SRX24215408 | SRS20985608 | S41 |
| SRX24215407 | SRS20985606 | S40 |
| SRX24215406 | SRS20985605 | S26 |
| SRX24215405 | SRS20985604 | S70 |
| SRX24215404 | SRS20985603 | S49 |
| SRX24215403 | SRS20985602 | S35 |
| SRX24215402 | SRS20985601 | S58 |
| SRX24215401 | SRS20985600 | S4 |
| SRX24215400 | SRS20985599 | S25 |
| SRX24215399 | SRS20985598 | S43 |
| SRX24215398 | SRS20985597 | S93 |
| SRX24215397 | SRS20985596 | S23 |
| SRX24215396 | SRS20985595 | S69 |
| SRX24215395 | SRS20985594 | S67 |
| SRX24215394 | SRS20985593 | S64 |
| SRX24215393 | SRS20985592 | S84 |
| SRX24215392 | SRS20985591 | S17 |
| SRX24215391 | SRS20985590 | S31 |
| SRX24215390 | SRS20985589 | S6 |
| SRX24215389 | SRS20985588 | S5 |
| SRX24215388 | SRS20985587 | S71 |
| SRX24215387 | SRS20985586 | S52 |
| SRX24215386 | SRS20985585 | S22 |
| SRX24215385 | SRS20985584 | S37 |
| SRX24215384 | SRS20985583 | S44 |
| SRX24215383 | SRS20985582 | S91 |
| SRX24215382 | SRS20985581 | S76 |
| SRX24215381 | SRS20985580 | S53 |
| SRX24215380 | SRS20985579 | S46 |
| SRX24215379 | SRS20985578 | S0 |
